# Country-level and individual correlates of overweight and obesity among primary school children: a cross-sectional study in seven European countries

**DOI:** 10.1186/s12889-015-1809-z

**Published:** 2015-05-08

**Authors:** Beatriz Olaya, Maria Victoria Moneta, Ondine Pez, Adina Bitfoi, Mauro Giovanni Carta, Ceyda Eke, Dietmar Goelitz, Katherine M Keyes, Rowella Kuijpers, Sigita Lesinskiene, Zlatka Mihova, Roy Otten, Christophe Fermanian, Josep Maria Haro, Viviane Kovess

**Affiliations:** Research, Innovation and Teaching Unit, Parc Sanitari Sant Joan de Déu (Universitat de Barcelona), Sant Boi de Llobregat, Spain; Instituto de Salud Carlos III, Centro de Investigación Biomédica en Red de Salud Mental (CIBERSAM), Madrid, Spain; EHESP Rennes, Sorbonne Paris Cité, EA 4069 Université Paris Descartes, Paris, France; Departmental House for Adolescents (Maison Départementale des Adolescents), Hautes-Alpes, France; The Romanian League for Mental Health, Bucharest, Romania; Liaison Pyschiatric Unit, Department of Public Health, University of Cagliari, Cagliari, Italy; Yeniden Health and Education Society, Istanbul, Turkey; Institute of Psychology, University of Koblenz-Landau (Campus Koblenz), Koblenz, Germany; Department of Epidemiology, Columbia University, New York, USA; Behavioural Science Institute, Radboud University Nijmegen, Nijmegen, Netherlands; Clinic of Psychiatry, Faculty of Medicine, University of Vilnius, Vilnius, Lithuania; New Bulgarian University, Sophia, Bulgaria; Netherlands Institute of Mental Health and Addiction (Trimbos Institute), Utrecht, Netherlands

**Keywords:** Overweight, Obesity, School children, Eastern and Western Europe, Individual and population-level correlates

## Abstract

**Background:**

The present study aims to estimate childhood overweight and obesity prevalence and their association with individual and population-level correlates in Eastern and Western European countries.

**Methods:**

Data were obtained from the School Children Mental Health in Europe, a cross-sectional survey conducted in 2010 in Italy, Germany, the Netherlands, Romania, Bulgaria, Lithuania and Turkey. The sample consists of 5,206 school children aged 6 to 11 years old. Information on socio-demographics, children’s height and weight, life-style and parental attitude were reported by the mothers. Country-level indicators were obtained through several data banks. Overweight and obesity in children were calculated according to the international age and gender-specific child Body Mass Index cut-off points. Multivariable logistic regression models included socio-demographic, lifestyle, mothers’ attitude, and country-level indicators to examine the correlates of overweight.

**Results:**

Overall prevalence was 15.6% (95% CI = 19.3-21.7%) for overweight and 4.9% (95% CI = 4.3-5.6%) for obesity. In overweight (including obesity), Romanian children had the highest prevalence (31.4%, 95% CI = 28.1-34.6%) and Italian the lowest (10.4%, 95% CI = 8.1-12.6%). Models in the pooled sample showed that being younger (aOR = 0.93, 95% = CI 0.87-0.97), male (aOR = 1.24, 95% CI = 1.07-1.43), an only child (aOR = 1.40, 95% CI = 1.07-1.84), spending more hours per week watching TV (aOR = 1.01, 95% CI =1.002-1.03), and living in an Eastern Country were associated with greater risk of childhood overweight (including obesity). The same predictors were significantly associated with childhood overweight in the model conducted in the Eastern region, but not in the West. Higher Gross Domestic Product and Real Domestic Product, greater number of motor and passenger vehicles, higher percentage of energy available from fat, and more public sector expenditure on health were also associated with lower risk for childhood overweight after adjusting for covariables in the pooled sample and in the east of Europe, but not in the West.

**Conclusions:**

Prevalence rates of overweight and obesity in school children is still high, especially in Eastern regions, with some socio-demographic factors and life-styles associated with being overweight. It is also in the Eastern region itself where better macro-economic indicators are related with lower rates of childhood overweight. This represents a public health concern that deserves special attention in those countries undertaking economic and political transitions.

## Background

A considerable body of research has grown concerning the prevalence of overweight and obesity in children from early ages to adolescence in a number of countries. Nevertheless, the literature seems to suggest that it is in the primary school where the highest prevalence of excess of weight is observed, with a peak at the age of 9 [1,2]. Moreover, interventions to prevent overweight and obesity seem to be most effective in this age group [3].

Despite many studies reporting the prevalence of overweight and obesity in school children, cross-national comparisons might be problematic due to some methodological issues [4]: the use of different criteria to classify children as overweight and obese [5], the variety of ages included, or how height and weight were measured [6]. Lobstein and Frelut [7] provided estimates of overweight and obesity in children aged 7–11 years old from nationally representative surveys of 21 European countries during the 1990s using the International Obesity Task Force (IOTF) criteria and anthropometric measures. Prevalence of overweight ranged from 10% in Russia to 35% in Malta. One limitation was the varying age ranges of the subjects and the years when the surveys were conducted for each country [8]. Existing research also suggests that the prevalence of overweight and obesity in school children is higher in Western Countries [7,8]. However, these studies were based on surveys conducted during the 1990’s [7] or early 2000’s [8] and this trend might have been inverted in the last years. Comparable data on school children from different European countries using the same survey and methodology are still needed to guide future health policies.

The literature also indicates that some risk factors for childhood overweight and obesity include those concerning the family and the sedentary lifestyle of the child; the socio-economic status of the family [9], number of siblings [10], rates of physical activity [8], and high levels of television viewing are associated with childhood overweight and obesity [11]. Despite the hypothesis that parental attitudes may also influence children’s eating habits [12], this topic is not fully understood. The identification of the role of parental behaviours might help in the design of effective treatment programmes for childhood overweight.

Modern environments may also be major contributors to current obesity trends [13]. Rabin et al. [13] found significant associations between several country-level indicators (e.g., Gross Domestic Product) and the prevalence of obesity in the adult population. Studies of the link between overweight and obesity in school children and country-level indicators of macro-economy or transportation are scarce, despite the fact that these indicators could explain the differences observed between countries and regions.

In summary, comparable data from distinct countries are needed to update information on the proportion of overweight and obesity in school children and to establish consistent trends in the association with correlates at an individual and population level. The aims of this study are: 1) to compare the proportion of overweight and obesity in samples of school children aged 6 to 11 years in seven European countries from Eastern and Western regions; 2) to explore the association with socio-demographic, life-style and parenting variables; and 3) to examine the associations with country level indicators of life-style, economy, transportation, health expenditure and policy domains.

## Methods

### Sample

The School Children Mental Health in Europe (SCMHE) was a cross-sectional survey conducted in 2010 to collect and monitor children's mental health using the same methodology across European countries. It took information from primary school children aged 6 to 11 years old and one of their parents, typically the mother. The present study includes data from Italy (n = 687), Germany (East n = 219 and West Germany n = 216), the Netherlands (n = 593), Romania (n = 1,003), Bulgaria (n = 984), Lithuania (n = 1,018) and Turkey (n = 486). Details on sampling by country are provided elsewhere [14]. Briefly, primary schools were randomly selected in each participating country. Approximately, 45–50 schools were approached per country (a greater number of schools were approached in Germany and the Netherlands), with varying school participation rates from 6.5% (Netherlands) to 95.6% (Romania). Classes within a school were randomly selected, and approximately 48 children were randomly selected in each school. The exception was the Netherlands, where fewer schools participated and, consequently, more students were selected from each participating school. The Italian sample was drawn from the island of Sardinia whereas in the rest of countries primary schools could be selected from the whole country [14]. Parents received information and a consent form to return to the school; children were included if they were present at school unless the parent actively refused. The general response rate was 66.4%, with the highest rate found in Lithuania (90.9%) and the lowest in Italy (44%). Mothers were the main respondents (86.8%). Since they are usually the main caregivers who spend more time with the child [15] and in order to reduce heterogeneity, the present study focused only on information provided by mothers, resulting in a final *n* of 5,206 children.

All participating countries had support and authorisation from their governments (generally from the Ministry of Education or Health). Ethical approval was obtained from the following ethics committees: 1) the Republic of Bulgaria, Deputy Minister of Education, Youth and Science; 2) The German Ministry of Education, Science and Culture Mecklenburg-Vorpommern, the German State school authority Luneburg, and the German Ministry of Education and Culture of Schleswig-Holstein country; 3) the Italian ethic committee of the Association of European University Mediterranean ONLUS; 4) the Republic of Lithuania – Ministry of Education and Science; 5) the Netherlands Commission of Faculty Ethical Behavior Research (ECG); 6) the Bucharest School Inspectorate General Municipal; and 7) the Istanbul directorate of National Education.

### Assessments

Parents completed a self-administered questionnaire about their children and returned it in a postage-paid return envelope. Children’s weight and height were reported by mothers and used to calculate BMI (as weight/height^2^, in kg/m^2^). Mother-reported measures were used to reduce cost, time, resources, and to administer the survey tool by post [16]. The international age and gender-specific child BMI cut-off points were used to classify children as ‘normal weight’, ‘overweight’, and ‘obese’ [17].

Socio-demographic variables included: child’s age and gender, mother’s educational level, whether the mother was professionally inactive, number of children at home, current marital status, age of the mother and the father, and whether they lived in a rural or urban area. Lifestyle behaviours included: time spent on physical activity and watching TV/video tapes/DVD (number of hours per week). Parenting attitudes were assessed with the Autonomy (7 items) and Care (8 items) subscales from the Parent Behaviours and Attitudes Questionnaire (PBAQ) [18], with a 4-likert type scale answers (3 ‘most of the time’; 2 ‘often’, 1 ‘sometimes’, 0 ‘never’). Z-scores were calculated for each item, stratifying according to age (6–8 and 9–11 years old). The total score on each subscale was obtained by adding the z-scores (mean z-score on the caring dimension = 0.06, standard deviation, SD = 5.05, and mean z-score on autonomy = −0.04, SD = 3.79). Three categories were then created for each dimension: below average (if the z-score was below the mean - SD), average (if the z-score was within the limit of mean ± SD), and above average (z-score higher than the mean + SD).

Country-level correlates included: Gross Domestic Product per capita (GDP, expressed in USA dollars and defined as the per capita monetary value of all final goods and services produced in a country during a year) and Real Domestic Product per capita (RDP, expressed in purchasing power parity (PPP), adjusted to the relative domestic purchasing power of the national currency as compared to the USA dollar) in 2010 [19]; the average number of calories available per person per day (total amount of food available for consumption in kilocalories), the percentage of total energy available from fat, and the average amount of fruits and vegetables available per person per year (in kg) in 2009 [19]; the numbers of motor vehicles (including cars, buses, and freight vehicles but not two-wheelers) per 1,000 people and passenger cars (including motor vehicles other than motorcycles intended for the carriage of passengers and designed to seat no more than nine persons) per 1,000 people in 2007 [20]; public sector health expenditure (the sum of outlays for health maintenance, restoration or enhancement paid for in cash or in kind by government entities, such as the Ministry of Health, other ministries, state organisations, and social security agencies) as a percentage of total health expenditure and total government expenditure in 2010 [19]; and the number of policies and actions developed by the governments of the seven countries from 2000 to 2010, aiming to promote healthy nutrition, physical activity and prevent obesity. This figure is equal to the number of distinct policies adopted by the governments of each country, with national coverage and targeted at children (although other populations could also be included: adolescents, elderly people, etc.) [21]. All these country-levels indicators were selected to allow comparison between our results and those reported by Rabin et al. [13].

### Statistical analysis

Prevalence rates of overweight and obesity were calculated in each country and region (Eastern Europe, including Romania, Bulgaria, Turkey, Lithuania and East Germany, and Western Europe, including Italy, the Netherlands and West Germany). Since the frequency of the category ‘obese’ was low in some countries, the overweight and obesity categories were merged and the subsequent analyses were conducted using the variable overweight as binary. Distribution of socio-demographic variables by the presence or absence of overweight (including obesity) was provided. Comparisons were calculated with the Chi-square test for categorical variables and the U-Mann Whitney test for continuous variables. Multivariable models included the significant variables from the bivariate analysis plus those considered important from a theoretical point of view. The multiple logistic regression models were computed for the total sample, the Eastern and Western Europe. The Odds Ratios (ORs) shown in tables were adjusted.

Multiple logistic regression models were computed to study the association between overweight and country-level indicators. Each indicator was standardised by subtracting the sample mean from each country’s value and dividing by the sample SD. These were then introduced in the models as z-scores. Separate models were computed for each z-score indicator, controlling for the presence of other covariates (those from the multiple regression model specified above). Models were then computed for the pooled sample and in the Eastern and Western regions.

Complete information on overweight and obesity was missing in 14.7% (n = 763) of the sample; children with these missing values were more likely to live in a home with more than 3 children, the parents were more likely to live apart, the mother’s educational level was lower, parents were younger and were more likely to live in Turkey. Multiple imputation as implemented in the PROC MI procedure in SAS statistical software was used, assuming that data were missing at random. The procedure took into account the socio-demographics, lifestyle and family variables described above. A total of 10 datasets were used and the logistic regression results were combined by using the PROC MIANALYZE procedure in SAS statistical software. Descriptive and bivariate analyses presented here were based on complete cases (not imputed).

All statistical analyses were performed with SAS®, version 9.3 for Windows [22].

## Results

### Socio-demographic characteristics of the sample

There were significant differences between Eastern and Western regions in some demographic variables (Table 1). Missing values varied from 0.1% (number of children at home) to 16.7% (hours per week of sport/physical activity).

**Table 1:** **Sample characteristics in Western and Eastern Europe (2010)**

		**Total sample (%)**	**Eastern Europe (%)**	**Western Europe (%)**	**P value**
	*Total sample size*	**5206**	**3710**	**1496**	
**Age**	Years *mean (SD)*	8.6 (1.32)	8.7 (1.25)	8.4 (1.47)	<0.0001
**Gender**	Male (n = 2613)	50.2	50.1	50.5	0.760
	Female (n = 2592)	49.8	49.9	49.5	
**Number of children living at home**	1 (n = 1473)	28.3	30.7	22.5	<0.0001
	2 or 3 (n = 3085)	59.3	54.4	71.2	
	≥4 (n = 642)	12.3	14.8	6.4	
**Marital status**	Single/Never Married/Separated/Divorced/Widowed (n = 739)	14.9	17.3	8.9	<0.0001
	Married/Remarried/Cohabitation/Other (n = 4214)	85.1	82.7	91.1	
**Mother’s education**	High school not completed (n = 597)	13.2	17.1	2.1	<0.0001
	High school completed (n = 1676)	37.1	40.6	26.9	
	Continued after high school (n = 2245)	49.7	42.3	71	
**Age of mother**	Years *mean (SD)*	36.9 (5.71)	35.4 (5.4)	40.5 (4.7)	<0.0001
**Age of father**	Years *mean (SD)*	39.4 (6.15)	37.9 (5.91)	42.7 (5.3)	<0.0001
**Mother professionally inactive**	Yes (n = 1567)	32.9	37.3	21.7	<0.0001
	No (n = 3200)	67.1	62.7	78.3	
**Area**	Rural (n = 545)	12.9	36.5	52.9	<0.0001
	Urban (n = 3667)	87.1	63.5	47.1	
**PBAQ caring**	Average (n = 3299)	67.8	64.4	75.9	<0.0001
	Below average (n = 824)	16.9	17.2	16.3	
	Above average (n = 745)	15.3	18.4	7.8	
**PBAQ autonomous**	Average (n = 3098)	64.5	65.5	62	<0.0001
	Below average (n = 851)	17.7	12.8	29.3	
	Above average (n = 855)	17.8	21.7	8.6	
**Sport/physical activity**	Hours per week *mean (SD)*	6.06 (7.43)	6.28 (8.28)	5.63 (5.36)	0.0031
**TV/video tapes/DVD**	Hours per week *mean (SD)*	6.81 (6.00)	7.16 (6.68)	6.05 (4.00)	0.962
**Overweight**	No Overweight (n = 3533)	79.5	75.5	88.1	<0.0001
	Overweight (n = 910)	20.5	24.5	11.9	

*Note:* Eastern Europe includes Romania, Turkey, Bulgaria, Lithuania, and East Germany; Western Europe includes West Germany, Netherlands and Italy.

### Prevalence of overweight and obesity

**Figure 1:**
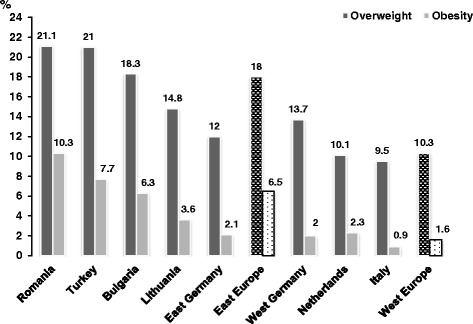
Proportion of overweight and obesity among school children by country and region.

The prevalence of overweight, including obesity, in the total sample was 20.5% (95% CI = 19.3 – 21.7%), 15.6% for overweight (95% CI = 14.5 – 16.6%) and 4.9% for obesity (95% CI = 4.3 – 5.6%). Figure 1 shows the prevalence of childhood overweight and obesity by country and region. Romania had the highest proportion of overweight (including obesity) (31.4%, 95% CI = 28.1-34.6%) and Italy the lowest (10.4%, 95CI% = 8.1-12.6%). In the Eastern region, the prevalence of overweight was 18% (95% CI = 16.6 – 19.4%) and 6.5% (95% CI = 5.6 – 7.4%) for obesity, while in the Western region it was 10.3% (95% CI = 8.7 – 11.9%) for overweight and 1.6% (95% CI = 0.9 – 2.2%) for obesity. The proportion of overweight and obesity in Eastern Europe was significantly greater than that in the West (χ^2^ = 102.97, p < 0.0001).

### Individual correlates of overweight

In the total sample, the children with overweight problems were more frequently boys, were only children, their mothers’ educational level was low, their mothers and fathers were younger, they were living in Romania or Bulgaria, and their mothers reported frequent unguided behaviours (Table 2). In Eastern Europe, children with overweight were mostly males, lived in a home with an only child, the educational level of their mothers was lower, their fathers were younger, and were more likely to be living in rural areas. Romania was the country with the highest proportion of overweight problems, followed by Turkey, Bulgaria, Lithuania, and East Germany. Sociodemographically, significant differences between overweight and normal weight children were not observed in Western Europe.

**Table 2:** **Distribution of demographic, lifestyles and family variables by presence of overweight problems**

		**Total sample**			**Eastern Europe**			**Western Europe**		
		**No Overw (%)**	**Overw (%)**	***p*** **value**	**No Overw (%)**	**Overw (%)**	***p*** **value**	**No Overw (%)**	**Overw (%)**	***p*** **value**
**Age**	Years *mean (SD)*	8.7 (1.33)	8.6 (1.28)	0.16	8.8 (1.23)	8.7 (1.23)	<0.01	8.4 (1.46)	8.4 (1.47)	0.931
**Mother’s age**	Years *mean (SD)*	37.4 (5.60)	36.3 (5.63)	<0.0001	35.7 (5.31)	35.3 (5.34)	0.095	40.5 (4.68)	40.5 (4.89)	0.932
**Father’s age**	Years *mean (SD)*	39.9 (6.05)	38.6 (6.12)	<0.0001	38.1 (5.81)	37.5 (5.77)	0.018	42.8 (5.26)	42.7 (5.71)	0.356
**Sport/physical activity**	Hours per week *mean (SD)*	6.2 (7.78)	5.9 (6.65)	0.056	6.5 (8.99)	6.1 (7.02)	0.235	5.7 (5.44)	5.3 (5.06)	0.214
**TV/video tapes/DVD**	Hours per week *mean (SD)*	6.8 (5.60)	7.5 (6.93)	0.264	7.2 (6.50)	7.7 (7.38)	0.221	6.0 (3.90)	6.3 (4.57)	0.902
**Gender**	Boys	48.9	53.6	0.010	47.5	55.9	<0.0001	51.4	43.7	0.06
	Girls	51.1	46.4		52.5	44.1		48.6	56.3	
**N° children at home**	1	26.5	34.4	<0.0001	28.3	36.9	<0.0001	23.1	23.4	0.673
	2 or 3	61.9	55.4		57.0	51.5		71.0	72.5	
	≥4	11.6	10.2		14.6	11.6		5.9	4.2	
**Marital status**	Single/Never married/Separated/Divorced/Widowed	14.1	14.9	0.546	17.1	15.7	0.384	8.4	11.3	0.237
	Married/Remarried/Cohabitation/Other	85.9	85.1		82.9	84.3		91.6	88.8	
**Mother’s education**	High school not completed	10.1	15.5	<0.0001	13.9	18.0	0.019	2.1	2.3	0.180
	High school completed	35.5	38.9		40.0	39.9		25.8	33.3	
	Continued after high school	54.3	45.7		46.1	42.0		72.1	64.4	
**Mother inactive**	Yes	11.0	14.8	0.004	15.2	17.0	0.301	3.9	6.1	0.205
**Area**	Rural	40.6	42.8	0.223	36.6	39.1	0.028	51.7	59.3	0.072
**Country**	Bulgaria	18.0	22.9	<0.0001	27.8	28.0	<0.0001	-	-	
	Romania	15.5	27.5		23.9	33.6		-	-	
	Lithuania	19.6	17.3		30.2	21.1		-	-	
	Turkey	7.1	11.1		10.9	13.6		-	-	
	East Germany	4.7	3.3		7.2	3.6		-	-	
	Italy	17.4	7.8		-	-		49.7	42.5	0.108
	Netherlands	12.8	7.0		-	-		36.4	38.3	
	West Germany	4.9	4.1		-	-		14.0	19.2	
**PBAQ caring**	Average	69.3	67.1	0.268	64.9	65.8	0.913	72.2	73	0.417
	Below average	15.9	15.9		16.2	15.6		15.3	17	
	Above average	14.8	17.0		18.9	18.6		7.5	10	
**PBAQ autonomy**	Average	64.6	65.3	<0.001	66.5	65.6	0.621	61.2	65.6	0.100
	Below average	18.9	13.9		12.3	11.6		30.6	23.9	
	Above average	16.5	20.8		21.1	22.8		8.2	11.9	

*Note:* Eastern Europe includes Romania, Turkey, Bulgaria, Lithuania, and East Germany; Western Europe includes West Germany, Netherlands and Italy.

We explored whether two specific items on the PBAQ questionnaire Autonomy subscale (“permission to prepare a simple dish” and “permission to go shopping”) were associated with childhood overweight. A significant association was observed in both (p = 0.0042 and p = 0.0009, respectively). Six percent of children never had permission to prepare a simple dish and 14.3% did not have permission to go shopping. The lowest prevalence of the item “having permission to prepare a simple dish most of the time" was observed in Italy (8.9%), whereas in other countries this proportion exceeded 30%, with the highest percentage reported in Lithuania (51.2%). Italian children had also less permission from their mothers (category "never") to prepare a dish. Similar results were observed in the variable "permission to go shopping".

The association between overweight and “permission to prepare a dish” was only significant in Italy (p = 0.0283). No significant associations were observed between “permission to go shopping” and overweight in any country.

Multivariable regression models in Table 3 showed that being younger (aOR = 0.93, 95% CI = 0.87-0.97), male (aOR = 1.24, 95% CI = 1.07-1.43), an only child (aOR = 1.40, 95% CI = 1.07-1.84) and spending more hours per week watching TV (aOR = 1.01, 95% CI = 1.002-1.03) were associated with being overweight. Compared with children in Italy, children living in Romania (aOR = 3.80, 95% CI = 2.77-5.21), Bulgaria (aOR = 2.72, 95% CI = 1.96-3.76), Lithuania (aOR = 2.05, 95% CI = 1.46-2.89), Turkey (aOR = 2.85, 95% CI = 1.94-4.19), and West Germany (aOR = 1.64, 95% CI = 1.03-2.61) had greater risk to be at greater risk of having overweight problems.

**Table 3:** **Association between socio-demographic, lifestyles and parenting attitudes with childhood overweight**

		**Total sample**	**Eastern Europe**	**Western Europe**
		***aOR (95% CI)***	***aOR (95% CI)***	***aOR (95% CI)***
**Age**		0.93 (0.87 - 0.97)*	0.92 (0.85 - 0.98)*	0.97 (0.86 - 1.1)
**Gender**	Male	1.24 (1.07 - 1.43)**	1.38 (1.18 - 1.63)***	0.76 (0.55 - 1.07)
	Female	1 [reference]	1 [reference]	1 [reference]
**N° children at home**	1	1.40 (1.07 - 1.84)*	1.39 (1.03 - 1.86)*	1.55 (0.64 - 3.75)
	2 or 3	1.09 (0.85 - 1.39)	1.05 (0.81 - 1.36)	1.40 (0.63 - 3.08)
	≥4	1 [reference]	1 [reference]	1 [reference]
**Level education mother**	High school not completed	1.27 (0.95 - 1.71)	1.27 (0.93 - 1.75)	0.93 (0.29 - 2.93)
	High school completed	1.08 (0.89 - 1.29)	1.03 (0.83 - 1.29)	1.27 (0.84 - 1.92)
	Continued after high school	1 [reference]	1 [reference]	1 [reference]
**Age mother**		1.001 (0.99 - 1.02)	0.99 (0.98 - 1.02)	1.01 (0.97 - 1.05)
**Country**	Romania	3.80 (2.77 - 5.21)****	2.67 (1.69 - 4.22)****	-
	Turkey	2.85 (1.94 - 4.19)****	1.98 (1.18 - 3.33)*	-
	Bulgaria	2.72 (1.96 - 3.76)****	1.92 (1.24 - 2.98)**	-
	Lithuania	2.05 (1.46 - 2.89)****	1.44 (0.92 - 2.27)	
	East Germany	1.42 (0.86 - 2.36)	1 [reference]	-
	West Germany	1.64 (1.03 - 2.61)*		1.62 (0.97 - 2.70)
	Netherlands	1.33 (0.92 - 1.92)		1.25 (0.83 - 1.90)
	Italy	1 [reference]		1 [reference]
**Sport/physical activity (hours per week)**		0.99 (0.98 - 1)	0.99 (0.98 - 1.002)	0.98 (0.95 - 1.02)
**TV/video tapes/DVD (hours per week)**		1.01 (1.002 - 1.03)*	1.01 (1.001 - 1.03)*	1.02 (0.98 - 1.06)
**PBAQ autonomy**	Below average	0.81 (0.65 - 1)	0.82 (0.63 - 1.06)	0.80 (0.53 - 1.21)
	Above average	1.13 (0.92 - 1.38)	1.09 (0.89 - 1.35)	1.40 (0.79 - 2.47)
	Average	1 [reference]	1 [reference]	1 [reference]

*Note:* Eastern Europe includes Romania, Turkey, Bulgaria, Lithuania, and East Germany; Western Europe includes West Germany, Netherlands and Italy.

In bold, significant association; *p < 0.05; **p < 0.01; ***p < 0.001; ****p < 0.0001. Adjusted Odds Ratios (aOR) and their 95% Confident Intervals based on a multivariable logistic regression that included all variables in the table.

Results were similar in Eastern Europe: school children who were younger (aOR = 0.92, 95% CI = 0.85-0.98), male (aOR = 1.38, 95% CI = 1.18-1.63), only children (aOR = 1.39, 95% CI = 1.03-1.86), and living in Romania (aOR = 2.67, 95% CI = 1.69-4.22), Turkey (aOR = 1.98, 95% CI = 1.18-3.33), or Bulgaria (aOR=1.92, 95% CI=1.24-2.98), compared with those living in East Germany, were more likely to be overweight. However, these associations were not observed in Western Europe.

### Country-level correlates of overweight

A description of the indicators by country, region and overall sample is provided in Table 4. In the overall sample, greater GDP (aOR = 0.69, 95% CI = 0.63-0.76) and RDP (aOR = 0.69, 95% CI = 0.63-0.76), the total % of energy available from fat (aOR = 0.71, 95% CI = 0.65-0.77), the number of motor vehicles (aOR = 0.68, 95% CI = 0.62-0.74) and passenger cars per 1,000 people (aOR = 0.69, 95% CI = 0.63-0.75), and public sector expenditure on health as a percentage of total government expenditure (aOR = 0.74, 95% CI = 0.68-0.81) were all associated with a reduced risk for childhood overweight (Table 5). The same indicators were also found to be significantly associated with lower risk of childhood overweight in Eastern Europe, but not in Western Europe.

**Table 4:** **Description of the indicators according to country, region and total sample**

**Indicator**	**Year** ^*^	**Unit**	**Romania**	**Turkey**	**Bulgaria**	**Lithuania**	**Germany** ^**†**^	**Netherlands**	**Italy**	**Eastern Europe**	**Western Europe**	**Total sample**
			**(n = 1,003; 19.3%)**	**(n = 486; 9.3%)**	**(n = 984; 18.9%)**	**(n = 1,018; 19.6%)**	**(n = 435; 8.4%)**	**(n = 593; 11.4%)**	**(n = 687; 13.2%)**	**mean (SD)**	**mean (SD)**	**mean (SD)**
**Gross domestic product (GDP)**	2010	US$ per capita	7670.3	10049.8	6334.68	11046	40163.8	46622.9	33786.6	10472.1 (7670.2)	39795.6 (5924.6)	18898.5 (15103.8)
**Real domestic product (RDP)**	2010	PPP$ per capita	14778.3	15829.8	13892.2	18120	37651.6	41673.2	32109.6	16948.2 (5440.6)	36700.7 (4429.9)	22624.3 (10326.9)
**Average number of calories**	2009	kcal/person/day	3487	3666	2791	3486	3549	3261	3627	3329.2 (328.7)	3470.7 (171.9)	3369.9 (299.3)
**Available fat**	2009	% of total energy	27.62	25.97	32.7	27.93	37.05	37.53	39.21	29.4 (3.1)	38.2 (0.9)	31.9 (4.8)
**Available fruits/vegetables**	2009	kg/person/year	226.6	361.2	105	171.9	176.2	212.6	312.4	194 (78.8)	253.2 (55.9)	211 (77.7)
**Public sector health expenditure (total health expenditure)**	2010	% of total health expenditure, WHO estimates	80.36	74.8	55.7	72.9	76.78	84.82	77.62	70.8 (9.5)	80.4 (3.6)	73.6 (9.3)
**Public sector expenditure on health (total government expenditure)**	2010	% of total government expenditure, WHO estimates	11.92	12.8	11.28	12.56	18.52	20.08	14.72	12.4 (1.6)	17.4 (2.5)	13.9 (3)
**Motor vehicles**	2007	N° motor vehicles/1000 people	180	131	295	479	623	503	677	314.7 (156.9)	583.2 (67.5)	391.8 (183.4)
**Passenger cars**	2007	N° passenger cars/1000 people	156	88	257	470	566	441	601	286.3 (158.4)	521.5 (66.3)	353.9 (174.5)
**N° policies to prevent obesity in children**	2010	N° policies to prevent obesity in children from 2000 to 2010	5	2	7	4	6	11	3	4.9 (1.6)	6.6 (3.7)	5.4 (2.5)

*Note:* Eastern Europe includes Romania, Turkey, Bulgaria, Lithuania, and East Germany; Western Europe includes West Germany, Netherlands and Italy.

*Indicators were obtained for 2010. Otherwise, data from the most recent year available were used (i.e., 2009, 2007); ^**†**^East Germany (n = 219) and West Germany (n = 216).

**Table 5:** **Indicators associated with children’s overweight problems in the total sample and by regions**

	**Total sample**	**Eastern Europe**	**Western Europe**
**Indicator**	**aOR (95% CI)**	**aOR (95% CI)**	**aOR (95% CI)**
**GDP**	0.69 (0.63 - 0.76)****	0.82 (0.75 - 0.91)****	1.12 (0.92 - 1.36)
**RDP**	0.69 (0.63 - 0.76)****	0.81 (0.74 - 0.90)****	1.13 (0.93 - 1.38)
**Available calories**	0.95 (0.88 - 1.02)	0.99 (0.92 - 1.09)	0.93 (0.77 - 1.12)
**Available fat**	0.71 (0.65 - 0.77)****	0.89 (0.82 - 0.97)*	0.84 (0.68 - 1.01)
**Available fruits/vegetables**	0.95 (0.87 - 1.03)	1.07 (0.98 - 1.18)	0.83 (0.68 - 1.003)
**Motor vehicles per 1000 people**	0.68 (0.62 - 0.74)****	0.76 (0.69 - 0.84)****	0.99 (0.83 - 1.20)
**Passenger cars per 1000 people**	0.69 (0.63 - 0.75)****	0.77 (0.70 - 0.84)****	0.99 (0.82 - 1.18)
**Public sector health expenditure as % of total health expenditure**	0.96 (0.89 - 1.04)	1.07 (0.99 - 1.17)	1.03 (0.86 - 1.25)
**Public sector expenditure on health as % of total government expenditure**	0.74 (0.68 - 0.81)****	0.84 (0.76 - 0.92)***	1.15 (0.95 - 1.40)
**N° of policies from 2000-2010**	0.97 (0.90 - 1.05)	1.02 (0.94 - 1.12)	1.10 (0.91 - 1.34)

*Note:* Eastern Europe includes Romania, Turkey, Bulgaria, Lithuania, and East Germany; Western Europe includes West Germany, Netherlands and Italy.

In bold, significant association; *p < 0.05; ***p < 0.001; ****p < 0.0001. GDP = Gross Domestic Product; RDP = Real Domestic Product. Adjusted Odds Ratios (aOR) and their 95% Confident Intervals based on logistic regression models. Each indicator was introduced as z scores. Models for the overall sample included z-scores calculated according to the means and SD for the overall sample; models for Eastern Europe and Western Europe included z-scores calculated with the mean and SD of each region, respectively. All models included one indicator and were adjusted for the presence of children’s age, gender, mother’s educational level, mother’s age, sport and physical activity (hours per week), TV/video tapes/DVD (hours per week), and PBAQ autonomy dimension.

### Sensitivity analysis

Multivariable logistic regression models were repeated in the pooled sample, and in Eastern and Western regions using complete cases. Results were similar to those obtained using the multiple imputation technique, except for age and number of hours per week spent watching TV. When using complete cases, neither of these predictors was significantly associated with childhood overweight although their ORs approached significance.

## Discussion

The overall prevalence of overweight, including obesity, in our sample of school children was 20.5%; 15.6% for overweight and 4.9% for obesity. One of the most striking results is the clear difference between Eastern and Western Europe: the proportion of overweight and obesity was 24.5% in Eastern samples and 11.9% in the Western regions, with Romanian children having the highest prevalence of overweight problems (31.4%) and Italians the lowest (10.4%). Further, this is the first study that attempted to look at the association between country indicators and overweight in school children. In line with the Rabin et al. study [13], our results showed that children living in countries with higher GDP and RDP, more motor vehicles and greater health expenditure had lower risk for overweight and obesity. Higher incomes are related to healthier dietary patterns [23] and to the health status of a country (i.e., lower prevalence of obesity) [24].

In the present study, the lowest proportion of overweight was observed in the Italian sample, whereas in Lobstein and Frelut’s review [7], the highest prevalence corresponded to Italy and Spain (South-Mediterranean countries). This could be explained in the light of the different methods used in the studies; Lobsteins and Frelut’s only included those studies that used anthropometric measures but not parent-report height and weight. In our study, prevalence of overweight could be underestimated, although in the rest of countries (e.g., Netherlands) the prevalence rates are quite similar to those reported in their review [7]. It should also be noted that in our study, the Italian subsample is representative of the island of Sardinia, which is roughly representative of Italy, despite considerable national north/south diversity. This could partially explain the differences observed between the present results and previous studies in terms of prevalence of overweight in Italian school children.

The risk for overweight (including obesity) differs between countries within the Eastern region: Compared with East Germany, Romanian, Turkish and Bulgarian children had 2.7 and almost twice the risk of being overweight, respectively. However, the risk of overweight did not differ among Western samples. Environmental and country-level characteristics might underlie these regional differences in Eastern countries, especially those concerning wealth (e.g., GDP) and the amount of money that governments devote to health. Conversely, one could expect greater homogeneity among Western Europe in terms of economic and health systems.

The percentage of overweight and obesity among school children is clearly higher in the samples from the Eastern region, compared to the West. The political and economic transition, and the socio-demographic changes in these countries might explain the growing ‘obesogenic niche’, which would include environmental factors that collectively predispose individuals to excessive weight gain [25]. All these changes might significantly affect the lifestyle and health status of the population, characterised by an increase in energy intake and a decrease in levels of energy expenditure [26]. Our results also indicate that it is in these Eastern regions where better macro-level indicators of economy, health expenditure and energy availability are significantly associated with better child health status.

In line with the findings of Rabin et al. [13], the prevalence of childhood overweight and obesity was lower in countries with high per capita availability of fat. This might be due to the fact that these type of data collected in the Eastern countries might not be representative or reliable [13]. It is also noteworthy that the relationship between dietary patterns and childhood overweight and obesity is still unclear. Children with overweight problems have been reported to be more likely to skip breakfast and avoid sweets and salty snacks [6]. The role of dietary patterns with respect to the risk of overweight and obesity in children and the interaction with other variables (e.g., physical activity) need to be determined in future studies.

Lower density of motor vehicles and passenger cars could indicate more active communities and be associated with lower rates of overweight and obesity. However, our results showed an inverse relationship. This could be because the number of cars might be also an indicator of the economic development of the country. Future studies should include other means of transportation and the built environment as predictors of childhood overweight (e.g., public recreation opportunities, fast-food outlets within the school) [27]. Our findings also support the notion that the health status of children (as expressed in lower prevalence of overweight) is better in those countries where expenditure devoted to public health is higher, thus urging governments to maintain and/or increase investment in policies intended to prevent overweight and obesity among school children.

As for individual correlates of children’s overweight, being younger, male, an only child, living in particular countries, and the number of hours per week that a child spends watching TV continued to be significantly associated with overweight after adjusting for other variables, in the total sample, and in Eastern Europe but not in Western Europe. Other studies on overweight and obesity in children have shown higher prevalence rates in younger children compared with older ones [7,28-30]. This decline in excess weight might be expected as children get older, since excess weight can be compensated for by growth [31]. Some studies have also demonstrated that boys are at greater risk for overweight than girls [6,32], and that this can be explained by risk factors for overweight and obesity possibly being gender-specific [33]. One could also argue that BMI might be related to more muscle mass and consequently explaining why boys, compared with girls (especially during the pre-pubertal period), have higher BMI.

In accordance with other studies [10,34], our findings show that children with no siblings are at higher risk of overweight and obesity. The role of siblings with respect to childhood overweight is unclear and requires further research. Other studies, for example, have found that having siblings is associated with higher consumption of soft drinks, sweets and snacks in young children [35-37]. However, the presence of other siblings might also stimulate play and therefore increase the time spent on physical activity [34]. The country of residence would reinforce this association, since families in Eastern countries are traditionally larger and the number of children at home could decrease the availability of food [10].

As found in other studies [38,39], those children who watched more TV had greater odds of being overweight, and this effect remained significant regardless of the child’s age, gender, other family and socio-demographic characteristics, and the number of hours doing sport. On one hand, watching TV for many hours might be associated with less physical activity and increased food intake in front of the television [40,41]. On the other hand, food intake might be higher while watching television because children are exposed to food-related stimuli such as food advertisements [40], and also because they are distracted and not aware of their consumption [42].

In our study, doing sport was not associated with overweight. It is possible that the immediate effect of exercise activities on food intake and preferences differ in normal weight and overweight children. For example, it has been shown that food and energy intake after some types of physical activity is much higher in children with overweight compared with non-overweight [43]. The association between physical activity and overweight in children warrants further research.

Despite the fact that our results did not show a significant association between mothers’ attitudes and children’s overweight in the multivariable regressions, there was a significant association between overweight and mother’s permission to prepare a simple dish only in the Italian sample. This might shed some light in the explanation of why the lowest prevalence of childhood overweight and obesity was observed among Italian children; Italian mothers’ control, on average compared with other countries, over what they children eat or buy might protect against the risk for overweight and obesity in the Italian children. These results suggest that cultural factors should be taken into account when designing successful preventive programs of overweight in school children, especially those involving parents’ attitudes.

There are some limitations to bear in mind. Prevalence rates of overweight and obesity might be underestimated because height and weight were based on mothers’ report [44]. However, some studies showed that parent-reported child height and weight were close to the corresponding measured means [45]. Moreover, assuming that the measurement bias is not differential among groups, this would not affect the results of statistical tests [16]. This study focused only on data reported by mothers, discarding those cases that were only reported by fathers. Mothers are typically the main informant of their children’s health status because they are usually the caregiver who spends most time with the child and can therefore provide more accurate information [15,46]. Mothers were the main informants in our study (86%), and this was done to reduce possible measurement errors. Despite the fact that schools, classes within a school and children were randomly selected in each country, the percentage of school participation was low in some countries (e.g., the Netherlands), and therefore, we cannot conclude that the findings are nationally representative. Moreover, the cross-sectional characteristic of the study prevents us establishing temporal associations with time-varying covariates. Other potential confounders were not included (e.g., mother’s and father’s obesity or peer influence) [47,48]. It should be emphasised that our results regarding indicators are limited to country-level associations, and do not take into account within-country variations and individual-level associations [13]. The sensitivity analysis showed that age and number of hours per week spent watching TV were not significantly associated with childhood overweight when using complete cases. This could be because the analysis of complete cases has limited power and may induce bias if missing values are related to covariates of interest, whereas under the missing-at-random assumption, multiple imputation corrects biases observed with complete case analysis [49] and increases power. Finally, the models for the effect of country-level indicators in the Western region might have yielded non-significant results as there was less variation due to fewer countries under study than in Eastern Europe.

## Conclusions

The prevalence of overweight and obesity in school children in 2010 was high and deserves attention from public health policy-makers. The difference between Eastern and Western regions is clear and might be explained in the light of the transition in the social, economic, and nutritional environments of the former ‘Eastern bloc’ countries [50]. Despite the prevalence of childhood overweight and obesity being lower in Western samples, such as Italy, Germany and the Netherlands, the proportion of childhood overweight and obesity is not negligible in these countries and should also be considered a health priority. The most striking finding of this study is the relationship between childhood overweight and obesity and indicators of the wealth of a country, especially expenditure devoted to public health. Finally, futures studies should examine more closely the interaction of lifestyles, built environment, sociodemographic, and other characteristics, in particular those concerning parental attitudes.
